# Who should provide care for patients receiving palliative chemotherapy? A qualitative study among Dutch general practitioners and oncologists

**DOI:** 10.1080/02813432.2018.1535264

**Published:** 2018-10-30

**Authors:** Jan Wind, Ineke C. Nugteren, Hanneke W. M. van Laarhoven, Henk C. P. M. van Weert, Inge Henselmans

**Affiliations:** a Department of General Practice, Academic Medical Centre Amsterdam, University of Amsterdam, Amsterdam, the Netherlands;; b Department of Medical Oncology, Academic Medical Centre Amsterdam, University of Amsterdam, Amsterdam, the Netherlands;; c Department of Medical Psychology, Academic Medical Centre Amsterdam, University of Amsterdam, Amsterdam, the Netherlands

**Keywords:** Primary Health Care, Medical Oncology, Interdisciplinary Communication, Palliative Care, Continuity of Care

## Abstract

**Introduction:** While close collaboration between general practitioners (GPs) and hospital specialists is considered important, the sharing of care responsibilities between GPs and oncologists during palliative chemotherapy has not been clearly defined.

**Objective:** Evaluate the opinions of GPs and oncologists about who should provide different aspects of care for patients receiving palliative chemotherapy.

**Design:** We conducted semi-structured interviews using six hypothetical scenarios with purposively sampled GPs (*n* = 12) and oncologists (*n* = 10) in the Netherlands. Each represented an example of a clinical problem requiring different aspects of care: problems likely, or not, related to cancer or chemotherapy, need for decision support, and end-of-life care.

**Results:** GPs and oncologists agreed that GPs should provide end-of-life care and that they should be involved in decisions about palliative chemotherapy; however, for the other scenarios most participants considered themselves the most appropriate provider of care. Themes that emerged regarding who would provide the best care for the patients in the different scenarios were expertise, continuity of care, accessibility of care, doctor–patient relationship, and communication. Most participants mentioned improved communication between the GP and oncologist as being essential for a better coordination and quality of care.

**Conclusion:** GPs and oncologists have different opinions about who should ideally provide different aspects of care during palliative chemotherapy. Findings raise awareness of the differences in reasoning and approaches and in current communication deficits between the two groups of health professionals. These findings could be used to improve coordination and collaboration and, ultimately, better patient care as results demonstrated that both disciplines can add value to the care for patients with advanced cancer.

**Key points**
- This study identified contrasting opinions of GPs and oncologists about who should provide different aspects of care for patients receiving palliative chemotherapy.
- Important themes that emerged were expertise, continuity of care, doctor-patient relations, accessibility of care, and communication.
- Although frequently using the same arguments, GPs and oncologists often considered themselves to be the most appropriate providers of palliative care.

## Introduction

The aim of palliative care is to improve the quality of life of patients and their families [1]. It is a major challenge to effectively coordinate this care because it is often provided by several health professionals working in primary and secondary care settings [2].

In the Netherlands, palliative chemotherapy is organized by hospital specialists, such as oncologists. Palliative chemotherapy is given in the non-curative setting to optimize symptom control, improve quality of life, and sometimes its given to improve survival. During this treatment phase, general practitioners (GPs) often lose contact with their patients and become involved again after chemotherapy has ended. This hampers the continuity and quality of care in the last phase of life [3]. In addition, communication between GPs and hospital specialists is often slow or inadequate, so GPs may not have up-to-date patient information [4]. In this phase, both patients and health professionals might become confused about who is responsible for different aspects of care [5]. This lack of clarity about who should provide different aspects of care for patients receiving palliative chemotherapy may have detrimental effects on the quality of care provided and might lead to more and unnecessary emergency department visits, inappropriate transfers, and unwanted dying in hospital [6,7].

Close collaboration between GPs and hospital specialists, with an increasingly greater role for GPs, is being promoted as a way to improve the quality of palliative care [8]. However, in a survey among European oncologists most of the respondents said that oncologists were best suited to provide palliative care for patients with advanced cancer, and considered themselves experts in the management of the physical and psychological symptoms of advanced cancer [9]. But on the other hand, nearly all oncologists expected primary care physicians to have a major role in all aspects of care in the palliative phase. Likewise, in another study 70% of primary care physicians reported being involved in the palliative phase [10].

To date, there is a lack of agreement about the respective roles and responsibilities of primary and secondary care during palliative chemotherapy. To improve collaboration and clarity on who should provide care, more insight is needed into differences in the approach and reasoning of GPs and oncologists with respect to various clinical problems in the palliative care trajectory. For this reason, the present study evaluated the views of GPs and oncologists about who should provide which aspects of care for patients receiving palliative chemotherapy in the Netherlands.

## Materials and methods

We conducted a qualitative study with semi-structured in-depth interviews using six hypothetical scenarios with purposively sampled GPs (*n* = 12) and oncologists (*n* = 10) in the Netherlands. We followed the COREQ criteria, a guideline for reporting qualitative research interviews.

### Design

One researcher and GP in training (IN) conducted all semi-structured interviews. Respondents were presented with six scenarios (see Table 1), each representing an example of a clinical problem requiring a doctor (a GP, an oncologist, or both) to take action. The use of different scenarios made it possible for the participants to express their views about the provision of different elements of care in a structured way. If necessary, they could refer to a case of their own. The scenarios were written by a GP (JW) and oncologist (HvL). The scenarios were based on observations about the role patients and oncologists assign to GPs during treatment planning or treatment evaluation in an outpatient oncology clinic published elsewhere [11].

**Table 1.** Description of the six scenarios discussed with the participated GPs and oncologists to express their views about the provision of different elements of care.

**Scenario 1** - Treatment of physical symptoms possibly related to the cancer or palliative chemotherapy:
Patient A, 48-year old female, diagnosed with metastasized breast cancer with disease progression on chemotherapy. In another hospital she had participated briefly in a phase 1 study, until a brain metastasis was diagnosed. She is on oral palliative chemotherapy (capecitabine). At the end of the second treatment cycle she developed respiratory symptoms without fever.
**Scenario 2** - Physical symptoms, most likely not related to the cancer or palliative chemotherapy:
Patient B, 70-year old male, diagnosed with metastasized colon cancer for which he underwent surgery and palliative chemotherapy. A recent CT scan showed treatment response. For a long time he has reported progressive pain in his right knee. He is worried that it is a metastasis.
**Scenario 3** - Pain management:
Patient C, 70-year old male, diagnosed with advanced pancreas cancer for which he is receiving palliative chemotherapy. The last few weeks he has experienced increasing abdominal pain; CT investigations have not found a cause. He was started on fentanyl in hospital and the dose was increased by the GP on call last weekend. The abdominal pain has got worse in the last few days.
**Scenario 4** - Treatment of physical side effects of palliative chemotherapy:
Patient D, 59-year old female, diagnosed with metastasized breast cancer that has progressed despite chemotherapy. She is currently on oral palliative chemotherapy (capecitabine). She has recently developed soreness, redness, and peeling on the palms of the hands (and soles of the feet), which causes pain.
**Scenario 5** – Decision support:
Patient E, 69-year old male, diagnosed with advanced pancreas cancer for which he receives palliative chemotherapy. He found treatment physically difficult. A recent CT scan has shown disease progression. The oncologist has offered him second-line chemotherapy within a clinical trial. The patient has been given information about the treatment, side effects, and prognosis. He is uncertain whether to have this treatment because of his poor physical condition and previous experience with the first-line chemotherapy and wants to discuss the situation with a health professional.
**Scenario 6** – End-of-life care:
Patient F, 39-year old female, diagnosed with metastasized breast cancer that has progressed despite chemotherapy. Because of her age and wish for treatment she has been given two different palliative chemotherapy treatments. She has developed brain metastasis, treated with radiation. The disease has progressed, causing multiple symptoms. As treatment has been discontinued, plans must be made for her care in the future.

### Participant selection and recruitment

GPs were recruited through the network of the department of General Practice of the Academic Medical Centre (AMC) in Amsterdam. All selected GPs worked in cities and villages of three provinces around Amsterdam, in the Netherlands. Oncologist were recruited from the hospitals in the working area of the participating GPs. The authors were not familiar with the participants. First, GPs and oncologists were invited by email with information about the study. Subsequently, they were informed by one researcher (IN, GP trainee) by phone on details about the study and asked for agreement to participate. During recruitment, we used purposive sampling to achieve a wide sample of participants with respect to gender, age, years of experience, for GPs area of occupation (i.e. urban vs rural) and for oncologist description of the hospital (i.e. academic vs non-academic). Details of the participants are shown in Table 2.

**Table 2.** Characteristics of participated GPs and oncologists in the Netherlands (*n* = 22).

	GPs (*n* = 12)	Oncologists (*n* = 10)
Age in years, mean (range)	47 (31–62)	46 (34–61)
Years’ experience, mean (range)	14 (0–35)	11 (0–25)
Gender, *n* (%)		
Male	6 (50%)	6 (60%)
Female	6 (50%)	4 (40%)
Description hospital/ practice, *n* (%)		
Non-academic		7 (70%)
Academic		3 (30%)
Urban	10 (83.3%)	
Rural (up to 20,000 inhabitants)	2 (16.7%)	
Number of days working per week, mean (range)	3.4 (1^a^–5)	4.7 (4–5)

^a^Also working in an out-of-office GP centre.

### Data collection

The semi-structured interviews were conducted face-to-face. The interview was pilot tested by IN (GP trainee). Inclusion continued until data saturation was achieved, defined as the situation in which no new codes appeared in three consecutive transcripts.

### Data analysis

Data were analysed using the six phases of thematic analysis described by Braun and Clarke [12]. One researcher (IN, GP trainee) checked transcripts against the original audio recordings for accuracy. After familiarizing themselves with the data, the coders performed the initial coding. Coding included identification of who should ideally see the patient per scenario (the GP, the oncologist, or both), pros and cons of a GP or oncologist as responsible health professional per scenario, and other interesting observations. IN independently coded all interviews and JW (GP) and IH (psychologist) each coded five interviews, so that ten interviews were double coded independently. Pairs of coders discussed their coding until agreement was reached. Coding was performed using software program MAX QDA version 11.0. The different codes were sorted into groups (for example, advantages and disadvantages of seeing a GP), to develop overarching themes. Results were compared and discussed by all coders. In the fourth phase, the potential categories identified in the previous phase were refined by IN, based on their validity in relation to the complete data set, making sure they accurately reflected the data. Fifth, the content of the groups and categories was analysed to generate clear definitions and names for each theme. Lastly, the results were reported, and appropriate quotes related to the research question and existing literature were selected.

## Results

**Figure 1.**
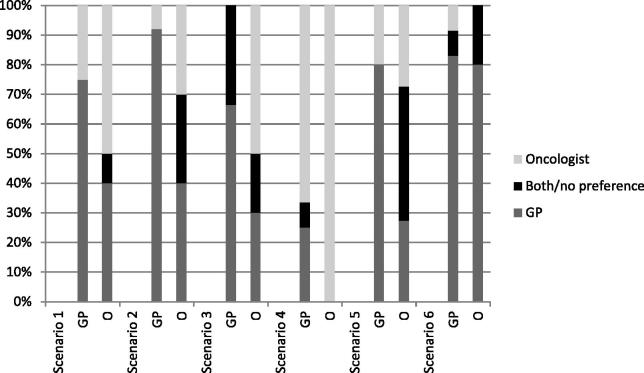
Who should be the ideal provider of care for patients receiving palliative chemotherapy according to the participated GPs and oncologists per scenario. Horizontal axis shows the participants: GP = general practitioner, O = oncologist. Vertical axis shows the ideal provider of care for patients receiving palliative chemotherapy: Both/no preference = the oncologist and the GP should see the patient or no preference.

Demographic information on the 22 participants is provided in Table 2. The interviews took on average 31 minutes (range 20–54 min). Two GPs had a special interest in palliative care, and two oncologists were part of the palliative care team in their hospital. Five major themes emerged from the analysis. Figure 1 shows who should ideally see the patient per scenario according to the participants. Although they often used the same arguments (Table 3), GPs and oncologists often considered themselves as being the ideal provider of palliative care. Table 4 shows participants’ quotes related to the arguments used in the interviews.

**Table 3.** Arguments derived from the interviews with GPs and oncologist about who should provide care for patients receiving palliative chemotherapy.

Who should provide care?	Pros and cons provided by GPs (*n* = 12) and oncologists (*n* = 10)
Oncologist	
+	More cancer specific knowledge (expertise)
	Part of cancer treatment (expertise)
	Can adjust treatment immediately (expertise)
	Retains overview (continuity)
	Frequent contact with patient (continuity)
	Easier access to additional diagnostic testing and results (accessibility)
	Provision of good transfer of end-of-life care to the GP (relationship)
	*Better doctor-patient relationship - oncologist (relationship)*
_	Not enough generalist knowledge (expertise)
	No continuity of care (continuity)
	Patient loses contact with GP (continuity)
	*Does not look at the context of the patient – GP (continuity)*
	*Difficult to provide customized patient care because of lack of network in neighbourhood - GP (continuity/accessibility)*
	More difficult to access and approach (accessibility)
	May perform unnecessary diagnostic / treatment (accessibility)
	*Hospital is a barrier to the patient - GP (accessibility)*
GP	
+	Has enough knowledge and expertise (expertise)
	Provides continuity of care (continuity)
	Better accessible and approachable (accessibility)
	Ability to keep in contact with the patient (continuity)
	Better overview of the patient / context (continuity)
	First-contact care/triage (accessibility/expertise)
	Can provide and coordinate care in home situation (accessibility)
	Serves as a mentor for the patient (relationship)
	Better doctor–patient relationship (relationship)
_	Insufficient knowledge and expertise (expertise)
	Much consultation needed with secondary care (expertise)
	Oncologist loses overview (continuity)
	Challenging to get (rapid) access to additional diagnostics and results (accessibility)
Both	
+	Patients’ choice
	Collaboration
	Different vision in whether to continue or stop the treatment
_	*Lack of advanced care planning (oncologist)*
	*Lack of overview of new prescribed medication (oncologist)*

The oncologist or the GP only mentioned the themes in italic. + is mentioned as an advantage by the participants. – is mentioned as a disadvantage by the participants. In parenthesis shows the corresponding theme.

**Table 4.** Quotes made by the GPs and oncologists on the six scenarios about who should provide care for patients receiving palliative chemotherapy.

	Who should see the patient?	
Scenarios	GP	Oncologist
**1. Physical symptoms, possibly related to palliative chemotherapy**	I can imagine if you experience these symptoms (running nose, cough, and no fever), you will first visit your GP – please listen to my lungs, do I need antibiotics? I mean, I think a GP would be able to assess this. **(quote of oncologist – GP provides first-contact care (triage))**	For us, it is easier to send someone to the lab or to take an X-ray of the lungs. Because when someone is neutropenic and they have an infection, you have to adjust the next chemotherapy session. **(quote of oncologist – oncologist has easier access to additional diagnostic testing and results)**
**2. Physical symptoms, most likely not related to the cancer or palliative chemotherapy**	I think we are better trained to deal with musculoskeletal problems. And we have more experience with their management. So if the problem is about mobility, I think we as GPs know what to do. **(quote of GP – GP has enough knowledge and expertise)**	The patient’s needs are leading. So, if someone feels ‘happy’ in the hospital and appreciates getting all his care there because they have to be there often. Then I think that’s fine. **(quote of GP – oncologist has frequent contact with patient)**
**3. Continuation pain management**	We would like to be told of new developments in the patient’s illness or complications of chemotherapy. And problems that are clearly not treatment related should be seen by the GP, because it is also good for the GP to keep in contact with the patient. **(quote of oncologist – ability for GP to keep contact with the patient)**	I would find it very frustrating if I were treating this man and the GP sent him, without consulting me, directly to a pain clinic. Because I will lose sight of the patient, and then I would wonder who is in charge of him. **(quote of oncologist - oncologist loses overview)**
**4. Treatment of physical symptoms, a side effect of the palliative chemotherapy**	No, well you know, most of the time the patient is tired of having to go to the hospital and if they can consult someone outside of the hospital, I do not mind at all. **(quote of GP - GP is better accessible and approachable**	I have no clue whether this is a result of chemotherapy. Looking at this symptom as a GP, I think what does the tongue look like, could it be a virus? Well, I have no idea. **(quote of GP – GP has not enough knowledge and expertise)**
**5. Decision support**	You have known the man for years and years, you know how he thinks about sickness and health (…) and especially when decisions have to be taken in a short time frame. Then I think it is really good, and valuable, if the patient can talk to his GP, whom he has known for several years. **(quote of GP - GP has better doctor-patient relationship)**	I think that it is difficult, because I have no knowledge of chemotherapy. Well, some of course but well, I do not know its exact pros and cons and the expected benefit. **(quote of GP- oncologist has more cancer specific knowledge)**
**6. End-of-life care**	When I think I cannot cure this anymore, I tell the patient to ensure that she has a good relationship with her GP, because she’s going to need the GP at some stage. I can treat the tumour to a certain extent, but those last 6 weeks at home, I will not be there. **(quote of oncologist – GP can evaluate and coordinate care in home situation)**	This has a lot to do with the relationship we build with patients over time. And then you are one step behind as a GP, which can give the patient the feeling they are getting dumped. So I always call them after a week or two. **(quote of oncologist - providing good transfer of end-of-life care to the GP)**

### Expertise (both general and cancer specific)

When discussing the scenarios, nearly all participants mentioned the importance of the cancer-specific knowledge of oncologists, and especially their knowledge of when and how to adjust chemotherapy, in contrast to GPs. This was mentioned more frequently when the participants thought that the patient’s symptoms and complaints were associated with chemotherapy. When this association was less obvious, as seen in scenario 2, predominantly GPs mentioned the lack of generalist knowledge of oncologists.

The participants, more often GPs than oncologists, repeatedly stated having sufficient trust in the knowledge and expertise of GPs. However, if a patient consultation was cancer related, fewer participants thought GPs would be sufficiently competent. In scenario 3, a third of the GPs said they lacked cancer-specific knowledge but they still wanted to remain in contact with the patient. In scenario 5, which was about decision support, all participants felt that GPs lacked expertise about chemotherapy, expected side effects, and influence on survival. In the last scenario about end-of-life care, only two GPs mentioned feeling not competent.

### Continuity of care

Continuity of care was considered important. Oncologists, but not GPs, mentioned that staying in charge helps them retain an overview of the patient in order to provide best care. For example, by staying informed about their medication use (scenario 3).

Mainly GPs mentioned that the continuity of care provided by GPs was essential, so that they can remain in contact with the patient throughout the cancer trajectory and during end-of-life care. The GPs also argued that they, unlike oncologists, could evaluate, coordinate, and adjust care to the patient’s home situation. In the last two scenarios about decision support and end-of-life care, particularly oncologists mentioned the advantages of the patient seeing their GP, such as seeing the patient ‘in real life’, involving family members in decision-making, and facilitating death at the preferred place (including euthanasia).

### Accessibility of care

GPs and oncologists mentioned that oncologists have easier access to additional diagnostic testing. This was mostly mentioned in the context of the first three scenarios. However, the GPs argued that easier access could lead to overdiagnosis and overtreatment. Half of the GPs and oncologists emphasized that GPs should provide first-contact care and perform triage. GPs and oncologists frequently mentioned the better accessibility (in terms of physical distance) and approachability of GPs, in contrast to oncologists. These arguments were mostly made in the context of the first two scenarios. Also both doctor groups mention that only the GP can perform home visits. This is a major advantage in the late stages of the disease. But also to provide first-contact and perform triage in patients who are not able to visit the GPs office or the oncologist in the hospital.

### Doctor–patient relationship (mentor)

Some oncologists mentioned that patients sometimes need the authority of a specialist to reassure them that their problems are not cancer related (scenario 2). In scenario 6 about end-of-life care, most of the oncologists and one GP mentioned it would be good for patients if they could still be in contact with their oncologist, so that oncologists could ensure a good transfer of care to the GP. Furthermore, remaining in contact with the oncologist might lower the threshold for going to the hospital, for example, if a blood transfusion is needed. This did not imply that oncologists should be the preferred doctor to consult.

Arguments for choosing GPs as preferred health professional included seeing the GP as a mentor, especially in scenario 5 about decision support. Participants emphasized that, in general, patients have a better and longer relationship with their GP. This makes it worthwhile to consult a GP when it comes to making difficult choices. Nevertheless, no remarks were made in the interviews on the influence of the length of the enlistment in the GPs office on the above mentioned aspects.

### Communication

All participants stated there were no protocols or agreements between oncologists and GPs regarding care provision and coordination. An issue that came up in all interviews was the poor communication between oncologists and GPs. Most participants emphasized that improving communication between colleagues is essential to improve the quality of care, especially in palliative care. The main topic addressed was the need to keep each other up-to-date about patient management, such as adjustment of medication (scenarios 3 and 4) and the transfer of care in the terminal phase (scenario 6). The most-mentioned barrier to communication was the number of doctors involved, sometimes in several hospitals, which could lead to essential knowledge and data being missed, for example, if the oncologist is not aware that the GP has increased pain medication. Some oncologists mentioned a lack of time for getting in touch with colleagues and the poor accessibility of GPs, especially during out of office hours.

GPs and oncologists emphasized that knowing which doctor is responsible for which patient and how to contact each other would improve communication. Participants frequently mentioned the importance of telephone contact, because that ensures that the person has received the information. Two oncologists mentioned email communication and working with electronic patient files, which they thought would create opportunities to obtain information without delay.

## Discussion

### Main findings

GPs and oncologists have different opinions about who should ideally provide different aspects of care during palliative chemotherapy. Although frequently using the same arguments, GPs and oncologists often considered themselves to be the most appropriate providers of palliative care issues. Important themes that emerged were expertise, continuity of care, doctor-patient relations, accessibility of care, and communication. Findings raise awareness of the differences in reasoning and approaches, and in current communication deficits between the two groups of health professionals. This could lead to better coordination and collaboration and, ultimately, better patient care as results demonstrated that both doctor groups are clearly needed.

### Literature

This study identified partially contrasting opinions of GPs and oncologists about who should provide different aspects of care for patients receiving palliative chemotherapy. This was best seen in scenarios about physical complaints during treatment, which might be related to the anti-cancer treatment, the cancer, or to neither. It would be helpful if doctors could agree on how to coordinate patient care in these types of scenarios.

Although frequently using the same arguments, GPs and oncologists often considered themselves to be the most appropriate providers of palliative care. This is in line with a recent study of psychosocial care in cancer survivors, in which both GPs and oncologists saw themselves as the main care provider [13]. We found that oncologists, as cancer experts, often felt responsible for dealing with the (somatic) consequences of cancer-related problems as well as the side effects of chemotherapy (scenarios 1–4). Most of the participating GPs emphasized that they would be willing and felt competent enough to provide care in most scenarios presented, both somatic as psychosocial). Sometimes the interviewed GPs expressed the need for the expertise of oncologists regarding the expected side effects or influence on survival of chemotherapy. In contrast to oncologists, GPs can serve as mentor by taking the whole context of the patient into account. This requires GPs to have a good doctor–patient relationship, which could be strengthened by involving GPs during the whole cancer trajectory, including contact for minor conditions. A previous study indicated that patients who know their GP better are more positive about their care [14].

In recent years, there has been growing interest in the role of GPs in the care of cancer patients. Studies have shown that, when using guidelines, GP-led care is not inferior to specialist-led care concerning quality of life and detecting recurrence [15,16]. Also, GP-led survivorship care is less expensive [15]. Although this debate largely focuses on follow-up care with a curative content for cancer patients, which is a different situation considering the presence of symptoms and crisis, but it is equally relevant to the role of GPs in a palliative setting. For example, when a recurrence is detected during follow-up, the GP can provide continuity of care.

Also, it is important to contemplate the needs of the patient – to what extent do they prefer a specific health professional, for example a GP, for the different aspects of care. We did not address this in our study. However, a systematic review reported that patients consider that GPs provide good care, especially when they take time to listen and discuss matters fully [17]. Additionally, in a survey among chemotherapy-treated patients, most participants reported that involvement of their GP was important to them. Yet, only 9% would consult their GP when an urgent problem would occur; 72% would turn to their oncology clinic. Most reported barrier mentioned to comprehensive care was lack of communication between treating teams [18].

Similarly, our study indicates that most participants emphasized that poor communication between oncologists and GPs hinders the provision of good-quality care. But to optimally use the skills and competencies of both oncologists and GPs, communication between the two groups of health professionals is essential. Previous research has also highlighted the deficits in communication and information transfer between health professionals in primary and secondary care [4]. When doctors involved in patient follow-up received a summary of the patient’s status and made early visits and timely follow-up calls, there were fewer unplanned readmissions [4,19]. Digital communication might be helpful in this regard, as suggested by our participants, although GPs still value direct communication with oncologists, for example, via phone or e-mail for specific urgent problems [20].

### Strengths and limitations

The strengths and limitations of this study need to be considered. The use of scenarios covering the different stages of cancer enabled us to interpret the results in a structured way and to create more robust key themes. On the other hand, a limitation of the use of specific scenarios may include that the crises were all around reactive care, but less about the ongoing review process which is also vital. Also, there were no real acute or out of hours scenarios highlighted. Furthermore, a scenario were a patient is currently not, or no longer fit to receive chemotherapy and is referred to the GP by the oncologist is missing. GPs often complain about unclear treatment decisions by oncologists. It would have been interesting to ask both GPs and oncologists who is responsible for a patient in this scenario. Finally, we did not vary the complexity of the systemic treatment in our scenario’s, which could have affected the findings.

Another limitation is that the interviewer works in primary care, which might have prompted participants, especially GPs to express socially desirable opinions. However, the observation that the participants mentioned both positive and negative arguments for both groups of health professionals suggests that they felt at ease discussing these issues. Another limitation is that we used quantitative data in order to visualize the differences between the opinions of the GPs and oncologists about who should be the ideal care provider in the six scenarios discussed (Figure 1). Furthermore, the way healthcare is organized in the Netherlands as well as the regional situation defines the structure of palliative cancer care. Our study was conducted in mainly urban situations covered by one or more hospitals. In the Netherlands the distance between the home of the patient and the GP and the hospital is in most cases limited. These factors limit the generalizability internationally. Finally, we did not assess the opinion of patients. The trust patients have in either their oncologists or GP may define which provider they prefer. In addition, practical considerations may play a role as well as the patients’ needs in specific situations.

## Conclusion

This study provides insight into the views of GPs and oncologists about current practice in care for patients receiving palliative chemotherapy. It shows partially contrasting perspectives on who ideally should provide different aspects of care. The findings raise awareness about differences in the reasoning of GPs and oncologists and in their approach to specific aspects of palliative care. In general, oncologists put more emphasis on topics such as understanding the prognosis and treatment options, whereas GPs focused more on the social network around the patient, knowledge of their past medical history, and their ability to keep an eye on the situation at home, which creates continuity of care. Awareness of these different points of view and of current communication and information transfer deficits between GPs and oncologist, should help to provide better individualized care for patients. Future research should look for methods to clarify the role distribution, to optimize interdisciplinary communication and jointly improve the quality of care for patients with advanced cancer.
